# Helpful and Hindering Factors in Psychodrama Field Training: A Longitudinal Mixed Methods Study of Student Development

**DOI:** 10.3389/fpsyg.2018.00196

**Published:** 2018-02-20

**Authors:** Bracha Azoulay, Hod Orkibi

**Affiliations:** School of Creative Arts Therapies, University of Haifa, Haifa, Israel

**Keywords:** psychodrama, students, professional development, identity, competence

## Abstract

Although the literature indicates that students in mental health professions start to form their professional identity and competence in graduate school, there are few studies on the in-training experience of creative arts therapies students. This mixed methods study examined how five first-year students in a psychodrama master’s degree program in Israel experienced their field training, with the aim of identifying the factors likely to promote or hinder the development of their professional identity and sense of professional ability. Longitudinal data were collected weekly throughout the 20-week field training experience. The students reported qualitatively on helpful and hindering factors and were assessed quantitatively on questionnaires measuring professional identity, perceived demands-abilities fit, client involvement, and therapy session evaluations. A thematic analysis of the students’ reports indicated that a clear and defined setting and structure, observing the instructor as a role model, actively leading parts of the session, and observing fellow students were all helpful factors. The hindering factors included role confusion, issues related to coping with client resistance and disciplinary problems, as well as school end-of-year activities that disrupted the continuity of therapy. The quantitative results indicated that students’ professional identity did not significantly change over the year, whereas a U-shaped curve trajectory characterized the changes in demands-abilities fit and other measures. Students began their field training with an overstated sense of ability that soon declined and later increased. These findings provide indications of which helping and hindering factors should be maximized and minimized, to enhance students’ field training.

## Introduction

Studies on the in-training experience of creative arts therapies (CAT) students are rare compared to those on student training in other healthcare professions. This is unfortunate since these studies can provide educators with valuable insights into student in-training needs and processes. The present longitudinal mixed methods study examined how five first-year students in a psychodrama master’s degree program in Israel experienced their first year of field training to pinpoint the factors likely to promote or hinder the development of their professional identity and sense of professional ability.

### Students’ Professional Development and Identity

Professional development has been defined as “an ongoing process through which an individual derives a cohesive sense of professional identity by integrating the broad-based knowledge, skills, and attitudes within psychology with one’s values and interests” (Ducheny et al., 1997, p. 89). There is relatively limited research on the professional development of CAT students, including in art therapy (Feen-Calligan, 2005), music therapy (Luce, 2008), dance movement therapy (Federman, 2011), and drama therapy or psychodrama (Orkibi, 2010, 2012a,c).

Orkibi’s (2014) two-year longitudinal study found that the Rønnestad and Skovholt (2003) seminal theory of counselors’ and therapists’ professional development was largely applicable to that of CAT graduate students in Israel. According to Orkibi, students in the Beginning Student phase (first year of a 2-year Master’s degree program) were mainly concerned with translating theory into practice, learning in a prescriptive and concrete way how experienced therapists practice, and reducing cognitive dissonance upon realization that their pre-training lay conceptions of helping were no longer valid. Stress and anxiety tended to prompt these students to adopt easily mastered techniques to implement in the practicum, and supervision was identified as the primary source of influence in this phase.

In the Advanced Students phase (second year), students began to critically assess their role models and accepted or rejected aspects they did not perceive as suited to their own perception of the therapist’s role. Advanced students developed a more complex view of client feedback and supervisor reactions which they used to gauge the effectiveness of treatment and by extension derive a sense of professional satisfaction. Generally, the findings suggested that students who were older and had undergraduate human-service education and/or considerable life experience were less concerned about their suitability to the profession, were more fully acquainted with a professional working style and sought to define their individual path toward becoming therapists.

The transformational process of professional development typically begins in graduate school and involves the formation of a professional identity (Kuther, 2008), which refers to identification with and emotional attachment to a given profession (Carson and Carson, 1998). In the CAT, students’ professional identity and career commitment were shown to be significantly correlated in the first semester of training (Orkibi, 2010) and increased during their 2-year training period (Orkibi, 2012a). A decrease was noted in students’ need for occupational and training information as well as the perceived environmental and personal barriers to career decision-making (Orkibi, 2012a). Feen-Calligan’s (2012) qualitative study identified a number of factors that fostered the professional identity of art therapy students, including a positive practicum and internship experiences, relationships with mentors, faculty and fellow students, courses taught by renowned art therapists, and experiential or art-based classroom assignments. Edwards (2015) suggested that becoming a music therapist emerges at an individual level (the student’s processing and learning), interpersonal (the dynamic between students as a group and with their trainers), and a broader meta-systemic level which includes the training institute, the faculty or department in which the training program is held, and the external regulating procedures of the state structures and the professional body (Edwards, 2015). In sum, the literature indicates that students’ formation of their professional identity starts in graduate school and is heavily dependent on self-reflection, an increasing sense of mastery and autonomy, and ongoing professional socialization. One important factor in the literature is students’ perceived demands-abilities fit.

### Perceived Demands-Abilities Fit

Studies on the determinants of educational and vocational success often refer to the *person-environment* fit framework, where “fit is defined as the degree of compatibility or match between an individual and the characteristics of his or her environment” (Nye et al., 2012, pp. 385–386). Environments can be an organization, job task, or academic major (Su et al., 2015). Academically, studies show that higher levels of fit (or congruence) between students’ vocational interests and their major is associated with better academic performance (Tracey and Robbins, 2006), persistence in academic major choice (Allen and Robbins, 2007), and a greater likelihood of timely degree completion (Allen and Robbins, 2010). Students’ perceived major fit correlated positively with academic self-efficacy (Wessel et al., 2008), satisfaction and GPA, and negatively with withdrawal intent (Schmitt et al., 2008). Nevertheless, the findings are mixed because some studies have reported only weak or no correlations with outcomes (e.g., Pozzebon et al., 2014).

In a recent CAT study, high scores on the Artistic and Social vocational types (i.e., exhibiting fit with the CAT profession that integrates arts and therapy) were hypothesized to negatively correlate with work burnout and positively with career commitment (Orkibi, 2016). The findings confirmed the significant role of person-environment fit in that students and professionals who were more self-expressive, creative, and original (artistic type) as well as more communicative, supportive, and interested in helping others (social type) were less vulnerable to the adverse effects of work burnout on career commitment than those who were lower on both of these vocational types.

The current study focused on a sub-domain of *person-environment* fit called the *demands-abilities* fit (D-A fit), which refers to the competence-related congruence between field training demands and student abilities as psychodramatists in training. Research shows that perceived D-A fit is positively correlated with employees’ organizational identification, job and career satisfaction, and occupational commitment, but not to job performance and raises (Cable and DeRue, 2002). In first-year undergraduates, perceived D-A fit was positively correlated with academic satisfaction, general life satisfaction, and academic performance and negatively correlated with major change intention and depression (Li et al., 2013). Perceived D-A fit was the strongest predictor of students’ subjective measure of academic performance, and D-A fit positively correlated with academic satisfaction and negatively with students’ intention to change their major (Etzel and Nagy, 2016). In a different study, major D-A fit positively correlated with GPA (Vahidi et al., 2016).

Overall, D-A fit thus appears highly consequential to students’ academic and personal functioning. The present study contributes to this literature by examining the understudied trajectory of perceived D-A fit of psychodrama students in field training, as well as the association between perceived D-A fit and their PI, perceived client involvement, and session quality. We predicted that students with a high D-A fit perception would also have high PI and would report high client involvement and session quality.

### Client Involvement and Session Quality

In this study we sought to quantitatively examine how psychodrama students’ perceived client involvement, session quality and in-session mood related to their sense of professional identity and perceived D-A fit throughout their first year of field training. Client involvement in sessions has been widely acknowledged as a common psychotherapy process factor that is important to session- and treatment-level outcomes across theoretical orientations (for a review see Morris et al., 2016). A moderate association between participation (encompassing involvement) and therapeutic outcome was found in a meta-analysis of 10 treatment studies with children and adolescents (Karver et al., 2006). Client involvement also significantly predicted a positive session evaluation as rated by both clients and therapists across different theoretical orientations (Eugster and Wampold, 1996). Related research on session evaluation has mostly looked at the client’s, rather the therapist’s, perceptions of session quality and its association with outcomes (Hill and Lambert, 2004; Chui et al., 2016). Although we were unable to locate studies with counseling or psychotherapy students focusing on our particular variables, it has been shown that higher levels of therapists’ negative mood *before* sessions associated with lower levels of therapist-rated helpfulness of their interventions and the quality of the sessions (Hill et al., 1994).

### Psychodrama Training

In most countries psychodrama training takes place in private rather than in academic institutions of higher education. In the United States, a prerequisite for psychodrama training to have a Master’s degree from an accredited university and postgraduate education in specified psychology/mental health areas. The psychodrama training itself is completed in certificate programs offered by private institutions by a certified psychodrama therapist who has a recognized TEP credential (i.e., trainer/educator/practitioner) awarded by the American Board of Examiners in Psychodrama^1^. In Europe, the Federation of European Psychodrama Training Organisations (FEPTO) has established “minimal training standards” that specify a Bachelor’s degree in specified psychology/ mental health areas as prerequisites for training. Psychodrama training is completed in FEPTO-recognized private institutions and to date, Master’s degree programs are only available in Austria, United Kingdom, and Israel^2^.

In Israel, the Council for Higher Education set down uniform standards in 2010 for graduate training in the CAT, including psychodrama. The prerequisites for training are an accredited Bachelor’s degree, 18 credits in specified psychology courses, and 500 h in the relevant art form (for psychodrama in drama or theater). According to the Council’s standards, a 2-year Master’s degree curriculum must consist of 40% psychotherapy courses and methodology courses and 60% CAT theoretical and experiential curses, seminars, and workshops in all modalities, as well as in the specific area of student specialization (e.g., psychodrama). With respect to field training, students must complete 600 h of supervised field training during the 2-year Master’s degree program and an additional 960 h of supervised post-Master’s advanced clinical training that is overseen by an accredited training program. Thus, students accumulate 1560 h of field training to qualify for a future (yet to be legislated) national exam and licensure by the Ministry of Health.

### The Present Study

The current study focused on students in a FEPTO-recognized Master’s degree training program that includes field training in health, welfare, rehabilitation, and education services with a variety of clients who have different problems. In the first year, field training takes the form of a guided clinical seminar and in the second year students are assigned to an independent practicum. This study focused on a clinical seminar, a weekly group field experience led and supervised by an experienced psychodramatist-supervisor. In the role of participant observers, students are required to pay attention to *how* and *when* they share themselves during a session, as well as *what* they share. When the students gain more experience they lead part of the session while the supervisor is also present. Thus, all students have several opportunities to take on a session leadership role, but their intervention is pre-approved by the instructor who closely monitors the intervention. The clinical seminar has four defining characteristics: (a) students are participant-observers in therapy sessions led by an experienced therapist, (b) the therapists closely monitors the students’ gradual therapeutic intervention with clients, (c) the therapists provide group supervision, and (d) students engage in milieu activities on the clinical site (see also Orkibi, 2012b).

The overarching research question in this study was how students experience their clinical seminar field training in terms of factors that are likely to promote or hinder the development of their professional identity and sense of professional ability. Qualitative changes in students were expected to be reflected in the quantitative measures collected. Thus, we also hypothesized that (1) students’ weekly scores on professional identity and D-A fit measures would increase throughout the year, and that (2) the increase in professional identity and D-A fit would be associated with increases in client involvement and session evaluation.

## Materials and Methods

We used a *concurrent triangulation* approach in a mixed methods design where qualitative and quantitative data were collected concurrently each week (Creswell, 2008). The secondary quantitative data were collected to assist in the interpretation of the primary qualitative data; namely, the quantitative data played a supportive role to the qualitative data by highlighting students’ trajectories on the quantitatively measured variables throughout the academic year. The concurrent triangulation approach is typically used to compare or relate two databases to determine whether there is convergence, divergence, or some combination of the two across the qualitative and quantitative data.

### Participants

Data were collected from five first-year students attending the same group of clinical seminar field training in the psychodrama Master’s degree program, ranging in age from 24 to 33 (*M* = 27). All students were born in Israel, four were Jewish and one was Christian; four were in a relationship or married and one was single. Two students had a Bachelor’s degree in theater/drama, two in social work, and one had a general degree in the behavioral sciences.

### Procedure

Data were collected throughout one academic year. The 20-week clinical seminar was held in a public junior-high school and involved the five students, seven 8th graders at risk and one Ph.D. level clinical instructor (CI) who has 15 years of experience as a qualified psychodramatist and 5 years of experience in facilitating this type of clinical seminar. The students and the CI adhere to the Code of Ethics of the Israeli Association for Psychodrama. Each week, on the day of their clinical seminar, the students received an email with a link to an online form. All the data were collected as parts of routine assignments during the clinical seminar. At the end of the academic year, after completion and grading of the clinical seminar, students were asked for permission to use their data in the study. It was clarified that participation was voluntary and that they had the right to refuse without penalty or prejudice to their interests. It was made clear that the data would be numerically encrypted. All students provided their informed consent that data can be used for research purposes.

### Measures

#### Demographics and Background Questionnaire

Data were collected on gender, age, country of birth, religion, marital status, number of children, place of residence, Bachelor’s degree, pre-training therapy experience as a client, and pre-training experience as a human service provider. In addition, we assessed student self-perceived dramatic competence in response to the following question: “Students come to psychodrama training from varying disciplines. Choose the answer that best represents your current view regarding your competence using tools from drama and theater (not necessarily in a therapeutic context). There is no right and wrong answer; an honest answer is most important.” Reponses were on a 4-point scale: 1 (*I think I am not at all competent*), 2 (*I think I am not so competent*), 3 (*I think I am quite competent*), 4 (*I think I am very competent*).

#### Helpful Aspects of Therapy (HAT)

Qualitative data were collected using the HAT form, a post-session open-ended self-report instrument that originally asked clients to identify and describe the most helpful/important and hindering events in the session in their own words (Llewelyn, 1988). The HAT form has been used to identify client perceptions of significant therapy events and clients’ narrative responses have been analyzed with a variety of qualitative methods (Elliott, 2010). In this study, the HAT form was slightly modified in that we clarified that “by ‘event’ we mean something that happened in the session. It might be something you said or did, something your CI or someone else in the group said or did, or a specific activity.”

#### Professional Identity

The 3-item career identity subscale of the three-dimensional career commitment measurement was used in this study (Carson and Carson, 1998). The two additional dimensions not measured here are career planning and career resilience. The original reference words “line of work/career field” were replaced with “psychodrama” as follows: “Psychodrama is an important part of who I am,” “Psychodrama has a great deal of personal meaning to me,” and “I strongly identify with the psychodrama profession.” Students rated the items on a scale ranging from 1 (*strongly disagree*) to 5 (*strongly agree*), where a high score reflects a higher sense of professional identity. The internal consistency reliability of this modified Hebrew version was very good with a Cronbach’s alpha of 0.83.

#### Demands-Abilities

The 3-item demands-abilities fit subscale of the three perceived fit scales was used in this study (Cable and DeRue, 2002). The two additional subscales not used here are person-organization fit and needs-supplies fit. Items with the original references to demands or requirements “of my job” were modified as follows: “The match is good between the demands on a psychodramatist and my personal skills,” “My abilities are a good fit with the requirements on a psychodramatist,” and “My personal abilities match the demands that the psychodramatist role places on me.” Students rated the items on a scale ranging from 1 (*strongly disagree*) to 5 (*strongly agree*), where a high score reflects a higher sense of demands-abilities fit. The internal consistency reliability of this modified Hebrew version was very good with a Cronbach’s alpha of 0.91.

#### Client Involvement

The 6-item Child Involvement Rating Scale used in this study included four positively worded items and two negatively worded items (Chu and Kendall, 2004). For this study, the term “child” was replaced by “participants.” Thus, instead of ranking each client’s individual level of in-session involvement, students rated the overall level of in-session involvement for all participants as a whole. A sample item is: “Participants demonstrated enthusiasm in therapy-related tasks.” The items were rated on a scale indicating the presence of involvement behavior in the group, from 0 (*not at all present*) to 5 (*a great deal present*) with higher scores representing higher involvement. Because recorded sessions for observational rating were not available for ethical reasons, after each session each student rated his or her perceived level of involvement in the session. The internal consistency reliability of this modified Hebrew version was good with a Cronbach’s alpha of 0.83.

#### Session Evaluation

To measure the students’ impression of each psychodrama session, the two sections of the Session Evaluation Questionnaire (SEQ; Stiles, 1980; Stiles et al., 1994) were modified and simplified in this study. As in Efraty (2007, Unpublished), our two sections measured *overall session* evaluation and *in-session mood* (instead of post-session mood in the original version). Each section had six bipolar adjective scales, with an opposite adjective on each end of the scale (instead of 10 items in each section). The SEQ session evaluation section measured students’ overall appraisal of the reported session quality where a higher score indicates better perceived quality. For each bipolar adjectives scale students were instructed to “mark the appropriate position on the scale that best represents this session for you.” The stem “This session was…” preceded the first six pairs of bipolar adjectives (e.g., bad-good, difficult-easy). The SEQ mood section measured students’ mood during the reported session, with a higher score indicating better mood. Students were instructed to “mark the appropriate position on the scale that best represents your feelings during this session.” The stem “During the session I felt…” preceded the second six pairs of bipolar adjectives (e.g., uncertain-definite, confident-afraid). In both sections, the scale was coded from 1 (for negative adjectives) to 5 (for positive adjectives). The mean score was calculated for each section. Internal consistency reliabilities of this modified Hebrew version were acceptable with Cronbach’s alphas of 0.79 for SEQ evaluation and 0.75 for SEQ mood.

### Data Analysis

#### Qualitative Analysis

Students’ narrative responses to the open-ended HAT form were first analyzed with a six-phase thematic analysis procedure (Braun and Clarke, 2006, 2012). The analysis sought to identify concepts and patterns within the data, primarily with respect to student perceptions of their own processes of development as therapists in-training. A cyclical analysis was continued until the point of saturation, when gathering more data seemed redundant, and no longer revealed new insights. Out of the five students, three reported 19 sessions, one reported 20 sessions, and one reported 18 sessions. All available sessions were analyzed.

#### Quantitative Analysis

We used a multilevel linear model (MLM) with the SAS PROC MIXED procedure (Jones and Huddleston, 2009), which enables the aggregation of single case results to the population level by taking into account a nested data structure (sessions nested in students). Because the visualization of outcomes over the sessions showed a parabolic trend (i.e., a curve similar to the shape of parabola), a quadratic session term was included in addition to the linear term. Using the same statistical framework (MLM), additional models were applied to test the relationships between the different measurements during treatment.

## Qualitative Findings

Thematic analysis of the students’ HAT forms yielded a longitudinal insight into their development over the academic year. The findings are presented according to three overarching themes: (a) change in perceived competence, (b) helpful factors, and (c) hindering factors. For each theme, the categories are described for the three phases throughout the academic year, which was divided according to trends that emerged in the quantitative data: beginning phase (sessions 1–5), middle phase (sessions 6–14), and culminating phase (sessions 15–19). A summary of students’ themes by phase is presented in **Table 1**. Pseudonyms are used for purposes of anonymity.

**Table 1:** Summary of students’ themes by phase.

Phase	Changes in competence	Helpful factors	Hindering factors
Beginning phase	Overstated sense of ability	-Clear and defined setting and structure -Observing the instructor as a role model	-Role confusion: Students as participant observers -Instructor as educator vs. therapist -Dealing with clients’ resistance and discipline
Middle phase	Self-doubts and confusion	Actively leading a warm-up	
			
Culminating phase	-More realistic view -Increased therapeutic thinking, language, reactions	Observing fellow students	School end-of–year activities

### A. Change in Perceived Competence

In the **beginning phase**, an overstated sense of ability was evident with respect to the students’ self-perceived demands-abilities fit. For example, one student stated: “Despite my fatigue and fear I felt I was as present and attentive as I wanted. This feeling made me feel more confident for the next meeting.” A different student wrote “I was successful in seeing what Avi (a client) really felt. It made me feel I am in the right place.” Another student reported “It was surprising to me how comfortable I felt during the session… quickly figuring out what to do in this new role.”

In the **middle phase**, the students’ overstatement of their professional suitability dropped; self-doubts and feelings of confusion, anxiety, uncertainty and distrust of their capabilities and their therapeutic skills emerged along with a significantly increased number of questions in their reports. For instance, a student remembered that “Ruthi (a client) said that she was searching on the Internet for a definition of psychodrama and found that it is treatment for children without friends.... I felt uncertain and had no words and was a little anxious that I had to contain it.” Another student was worried about the idea of being a group leader stating, “I wondered how I would cope as a group facilitator. There were moments when I asked myself whether the adolescents wanted to be here at all. Maybe they do not want to? Maybe we should terminate the group?” A different student shared “The big question I am struggling with is ‘what to do.’ What to do when they [the clients] sit like this, how to respond to Mira’s [the client’s] negativism or to unpleasant tones.”

In the **culminating phase** a more realistic professional capability emerged. For example one student said: “Today, unlike in the past, I understand that even when the group is stormy and noisy, work takes place.” Another student wrote about recognizing “opportunities” to identify therapeutic elements in the psychodramatic sessions: “My clinical understanding became sharper and clearer that it is also possible to use indirect dramatic activities … that can enable the client to freely express personal themes.” A different student stated that “Role playing enabled me to play with Tal (a client) and created an opportunity to strengthen and empower his self-confidence.”

In this culminating phase, the students also increasingly started to think more therapeutically as psychodramatists and to use psychodramatic and clinical professional terms: “I have now realized that it is very helpful to use simpler and more theatrical terms with adolescents rather than psychotherapy jargon.” Another student wondered: “how do I manage the countertransference more efficiently without ‘waiting’ for them to disappear by themselves?” A different student wrote: “distancing the role from ourselves, defining it as a part within us, showed me the strength of psychodrama role theory.” Another student pointed out that “role reversal enabled Dina (the client) to be more authentic with her words, body posture and tone of voice. I could see the expansion of her role repertoire as she didn’t take her typical role…”

Similarly, the students appeared to have learned to react therapeutically to a client’s “interruptions” in the group. This was reflected in the students’ reported ability to react more therapeutically to clients and to perceive disciplinary issues and maladaptive behaviors as psychological material to work with: “I was able to reflect to the client that he was very ‘stormy’ instead of saying that he is ‘interrupting the session’.” Another student reported: “When a client wanted to leave the room, I didn’t panic, I told her it would be better if she stayed and ‘told us why you are so angry, it might also represent other voices in the group’.”

### B. Helpful Factors

In the **beginning phase**, students highlighted components that were meaningful in orienting them to psychodrama field training. The most significant components were having a clear and defined setting and structure, such as a defined timeframe for the session, a defined session structure (working in large and smaller groups), clear goals for the group, an explicit contract, recurring pre-group arrangements of the room and materials, and a recurring opening ritual. One student stated: “First things first: the setting. It was helpful to all of us that when the girls and boys first entered the classroom, the chairs were arranged in a circle.” Other students felt it was “important that the instructor clearly presented the group’s goals in the opening session” and that “defining the group’s rules and norms, including confidentiality, seems to have provided a framework, an obligation and confidence to the boys and girls.” The students noted that the setting and structure enabled them and the clients to form relationships and become “more and more engaged and involved.” Introductory playful psychodrama activities and techniques were identified as “tools that made us more readily involved in the process.” Overall these findings suggest that these components contributed to the feeling of holding and contained environment in the group, for both the students and the clients.

Another helpful factor within the session was observing the CI in action who served as a role model. The students felt safer observing than actually doing. This is illustrated by the following statement: “It is interesting to observe how during your [the CI] conversation with Ella [the client], as an observer, to see you listening, thinking, and only then deciding together with her how to start the scene.” Another student stated: “The way in which you [CI] conducted yourself allowed me to be calm in the midst of the bustle and learn how to cope with the lack of cooperation and the resistance that arose.”

In the **middle phase**, one of the most helpful factors was the students’ own active participation in leading the session warm-up, “My experience in delivering the warm-up was the most significant for me, because I could experience the challenge of facilitating a group.” This hands-on role was illustrated in another way: “I’m glad I gave the warm-up today, I feel I have more confidence to stand in front of the group… I had a good learning experience. Especially the importance of improving the way I introduced the exercise instructions.” Another student noted “A meaningful event was my warm-up exercise before the action phase… I had the opportunity to self-introspect on how I act when I need to talk with a teenager, to expand and develop the dialog.”

In the **culminating phase** the key helpful factor was observation of fellow students, which seemed to reinforce their own competency: “I saw Liat’s warm-up [exercise] and I felt more relaxed today and beyond that, I knew that if my exercise did not go as I planned it would not be the end of the world.” Students’ understanding of the psychodramatist’s role was also clarified, specifically as regards being therapeutic, spontaneous, creative, “facilitating a contained and holding environment,” “mastering psychodrama theory, processes, and techniques according to the client’s needs.”

### C. Hindering Factors

The hindering factors in the beginning phase were similar to those in the middle phase and therefore are reported together. The main hindering factor was role confusion. There was confusion regarding students in the participant-observer role in that they often over-identified with the teens’ issues and did not have enough clinical experience to identify countertransference processes or to effectively manage it. They used lay terminology to describe their feelings and experience in the group. For example, one student wrote: “I know that my identification with his [the client’s] pain draws on times when I felt rejected in my own past.” When reflecting on her reaction to three boys who acted-out in the group, another student stated “I wonder if the boys recognized that they were triggering me? Could I have reacted differently to what they did in the group?” The second type of confusion had to do with the role of the CI (who was the group facilitator); students were confused about her role as educator as compared to therapist, given the fact that psychodrama group was taking place in a school. For example, one student stated he “felt offended for the instructor when the teens interrupted and acted-out rather than listening to her [the CI].”

Another hindering factor in the beginning phase involved dealing with clients’ resistance and disciplinary issues, which students viewed as impeding the group process. An illustrative example is the following: “During the session, some of the boys spoke among themselves in Russian and were also playing with their cell phones. This was after being asked politely several times to speak Hebrew and put the cell phones away.” Another illustration related to the disciplinary stance is reflected by a student who wrote “the three boys were late again and it creates a disruption when we need to wait for them. They are not well behaved…”

In the **culminating phase**, there were only a few reports of hindering factors, and student increasingly used professional terminologies more accurately. The most notable hindering factor was the school end-of-the-year activities which, according to students “invites rule breaking,” “disrupts the continuity and setting” and “interrupts the therapy process.”

## Quantitative Findings

### Hypothesis 1: Trajectories of Change in Students’ Scores

**FIGURE 1:**
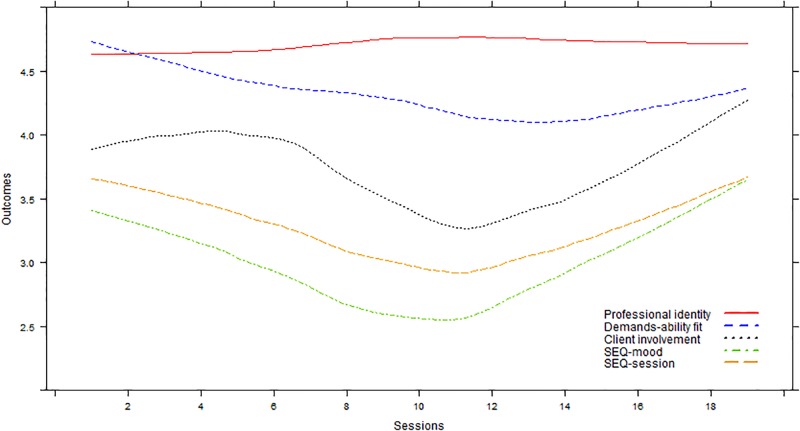
Trajectories of change in students’ scores over the course of field training sessions. SEQ, session evaluation questionnaire.

**Figure 1** depicts the patterns of change in student scores over sessions. The students’ professional identity did not change significantly over sessions (*p* = 0.057). However, a significant trend of change was found in students’ perceived D-A fit (*B* = 0.001, *t* = 2.71, *p* = 0.008), students’ perceived client involvement (*B* = 0.009, *t* = 2.39, *p* = 0.02), students’ general evaluation of the sessions (*B* = 0.07, *t* = 5.28, *p* < 0.001), and students’ evaluation of their mood during the sessions (*B* = 0.05, *t* = 4.07, *p* < 0.001). Note that the depiction of the outcomes over the sessions exhibited parabolic trends that have the tendency to initially decrease only slightly and then become steeper with time, often taking the form of a U-shaped curve.

### Hypothesis 2: Relationship between Variables

The results showed that students’ professional identity (inserted into the statistical model as an outcome variable) was significantly and positively correlated with students’ perceived client involvement (*B* = 0.11, *t* = 2.36, *p* = 0.02), students’ general evaluation of sessions (SEQ-session; *B* = 0.04, *t* = 3.46, *p* < 0.001), and students’ mood during sessions (SEQ-mood; *B* = 0.03, *t* = 2.60, *p* = 0.01). Students’ perceived D-A fit (inserted into the model as an outcome variable), did *not* correlate with any of the variables. The qualitative and quantitative findings are discussed below.

## Discussion

This longitudinal mixed methods study examined the experience of psychodrama students during their first year field training in a clinical seminar held in a public junior high school. Students were in the role of participant-observers in psychodrama sessions led by an experienced psychodramatist-supervisor. We aimed to identify factors that helped or hindered the development of their professional identity and sense of professional ability.

Taken together, the quantitative and qualitative findings suggest that students undergo a U-shaped curve trajectory during their first year of psychodrama field training. They began their field training with an overstated sense of ability that later declined and then increased. This trajectory generally corresponds to the pattern described in studies in developmental psychology which have indicated that whereas self-competency beliefs can be unrealistically positive in young children (Nicholls and Miller, 1984), these beliefs decline across middle childhood and early adolescence (Jacobs et al., 2002), probably as they start to make social comparisons (Suls et al., 2002).

Similarly, CAT students enter training with their own personal lay conceptions of helping, as they often see themselves as helpers to friends and family members where helping is typically guided by common sense and personal life experience (Orkibi, 2014). Students may consciously or unconsciously overestimate their helping abilities as a form of self-protection; in other words, they may strive to protect and enhance their feelings of self-worth (Shepperd et al., 2008). In fact, it has been suggested that CAT students’ self-serving cognitive bias may “reflect a need to defend their professional choice and ratify their emerging professional identity” (Orkibi and Bar-nir, 2015, p. 33).

As training progresses, some students start to engage in upward social comparison, when comparing themselves to the CIs or to a more experienced peer. Consequently they may feel more insecure about the fit between their abilities and the demands of the therapist role (Orkibi, 2014, p. 515). Thus, the subsequent quantitative decline in students’ sense of ability and the qualitative findings that reflect an increase in self-doubts and confusion regarding suitability are consistent with Orkibi’s (2014) study where first-year students experienced increased stress and anxiety upon the realization that their personal self-endorsed pre-training lay conceptions of helping others were no longer valid.

Surprisingly, students’ perceived professional identity remained stable over the year (it did not change significantly over sessions) possibly because the professional identity scale assesses a general identification with a chosen profession. In contrast, the four other scales that did indicate change (D-A fit, client involvement, and the two session evaluations) rely heavily on ongoing in-session occurrences and experiences. The reasons why the D-A fit did not correlate with client involvement and session evaluations should be explored in future studies with a larger sample. These relationships may have been affected by intervening variables (i.e., moderators) such as student pre-training experience in the care provider role as well as student feedback from the CI.

The findings are indicative of the helping and hindering factors that should be maximized and minimized to cultivate students’ successful field training. As in Orkibi (2014), the qualitative findings here suggest that students need a stable setting and clear group rules and that they rely on their CI (who led the group) as a viable professional role model. The findings also highlight the importance of gradually enabling students to take on a leading role in the group, under the close supervision of the CI. The results suggest that leading hands-on warm-up activities is a significant first step in rebuilding students’ sense of competence after their earlier experiences of doubt.

The findings on the hindering factors highlight the importance of making sure the students have a clear perception of their role as participant observers, which requires that they be mindful and prudent about sharing during the sessions. From a psychodramatic standpoint, the role of the participant-observer corresponds to that of the auxiliary ego: a group member who plays a role in the protagonist’s psychodrama and thus is an extension of the therapist in the service of the protagonist (Holmes, 1998). The key hindering factors were related to the school setting of the field training. The literature on school-based CAT indicates that one of the challenges that can influence the effectiveness of therapy itself and may impede the therapist’s work is role confusion (Karkou, 2010; Leigh, 2012), as noted by the students here. As was the case for our students, studies have noted the challenges associated with changes in the location of the therapy room and outside disruptions that interfere with the safe and familiar therapeutic space, as well as school holidays, activities, and excursions that interrupt the continuity and stability of therapy (Wengrower, 2001; Regev et al., 2015; Keinan et al., 2016; Belity et al., 2017).

## Conclusion

The strengths of this study include the longitudinal examination of the understudied trajectories of professional identity, perceived D-A fit, client involvement, and session evaluation of psychodrama students throughout field training. In addition it examined the association between these variables and the triangulation of qualitative and quantitative data in a mixed methods design constructed to offset the disadvantages of using one method with the advantages of another (Creswell, 2008).

However, there are several limitations that should be addressed in future work. Studies on students’ professional development during training would benefit from a lager sample size for the quantitative analysis. This would enable the use of more complex statistical procedures such as the examination of moderated and mediated relationships between variables. Second, the transferability of the findings is somewhat limited to field training with junior high school adolescents and field training with other age groups or in other settings (e.g., hospitals) could yield different results. Third, whereas this study involved psychodrama Master’s degree students, most psychodrama training programs in the world are run in private institutions. Nevertheless, the findings may be more applicable to field training in other CAT Master’s programs. In addition the reliance on students’ self-reports may have been influenced by social desirability bias. Future studies should not only measure social desirability (e.g., Fischer and Fick, 1993) but should also collect data on CAT students’ performance (e.g., D-A fit) from other sources such as students’ CIs in the field, university supervisors, and even peers. This data triangulation could be used to cross-validate students’ perceived competence as well as highlight the putative disparities between students’ perceptions and their actual abilities. Despite these limitations, we hope that this study will encourage others to further examine understudied in-training processes that can ultimately lead to the reconsideration and improvement of the clinical field training of future creative arts therapists.

## Ethics Statement

The study was exempt from ethical approval procedures because all the data were collected as parts of routine assignments during the clinical seminar. At the end of the academic year, after completion and grading of the clinical seminar, students were asked for permission to use their data in the study. It was clarified that participation was voluntary and that they had the right to refuse without penalty or prejudice to their interests. It was made clear that the data would be numerically encrypted. All students provided their written consent.

## Author Contributions

All authors listed have made a substantial, direct and intellectual contribution to the work, and approved it for publication. The authors contributed equally to this manuscript.

## Conflict of Interest Statement

The authors declare that the research was conducted in the absence of any commercial or financial relationships that could be construed as a potential conflict of interest. The handling editor is currently co-organizing a Research Topic with one of the authors, HO, and confirms the absence of any other collaboration.

## Footnotes

1 https://www.psychodramacertification.org

2 http://www.fepto.com/
